# Screening for QT Prolongation in the Emergency Department: Is There a Better “Rule of Thumb?”

**DOI:** 10.5811/westjem.2019.10.40381

**Published:** 2020-02-21

**Authors:** Megan L. Rischall, Stephen W. Smith, Ari B. Friedman

**Affiliations:** * Hennepin County Medical Center, Department of Emergency Medicine, Minneapolis, Minnesota; † University of Pennsylvania, Department of Emergency Medicine, Philadelphia, Pennsylvania

## Abstract

**Introduction:**

Identification of QT prolongation in the emergency department (ED) is critical for appropriate monitoring, disposition, and treatment of patients at risk for torsades de pointes (TdP). Unfortunately, identifying prolonged QT is not straightforward. Computer algorithms are unreliable in identifying prolonged QT. Manual QT-interval assessment methods, including QT correction formulas and the QT nomogram, are time-consuming and are not ideal screening tools in the ED. Many emergency clinicians rely on the “rule of thumb” or “Half the RR” rule (Half-RR) as an initial screening method, but prior studies have shown that the Half-RR rule performs poorly as compared to other QT assessment methods. We sought to characterize the problems associated with the Half-RR rule and find a modified screening tool to more safely assess the QT interval of ED patients for prolonged QT.

**Methods:**

We created graphs comparing the prediction of the Half-RR rule to other common QT assessment methods for a spectrum of QT and heart rate pairs. We then proposed various modifications to the Half-RR rule and assessed these modifications to find an improved “rule of thumb.”

**Results:**

When compared to other methods of QT correction, the Half-RR rule appears to be more conservative at normal and elevated heart rates, making it a safe initial screening tool. However, in bradycardia, the Half-RR rule is not sufficiently sensitive in identifying prolonged QT. Adding a fixed QT cutoff of 485 milliseconds (ms) increases the sensitivity of the rule in bradycardia, creating a safer initial screening tool.

**Conclusion:**

For a rapid and more sensitive screening evaluation of the QT interval on electrocardiograms in the ED, we propose combining use of the Half-RR rule at normal and elevated heart rates with a fixed uncorrected QT cutoff of 485 ms in bradycardia.

## INTRODUCTION

In the emergency department (ED), emergency providers encounter patients with prolonged QT for many reasons, including drug overdose, hypokalemia, hypomagnesemia, and therapeutic use of QT-prolonging medications. QT prolongation is a known risk factor for torsades de pointes (TdP). While TdP often self-terminates, it can be associated with hemodynamic instability and collapse and may degenerate into ventricular fibrillation and resultant cardiac death. Identifying ED patients with prolonged QT and risk of TdP is crucial to allow for appropriate monitoring, interventions, and disposition.

Unfortunately, computer electrocardiogram (ECG) algorithms are unreliable in identifying prolonged QT. Prior studies have shown that computer ECG algorithms are often inaccurate in measuring QT interval, particularly in abnormal or poor-quality ECGs. 1 Additionally, when these algorithms do identify prolonged QT, they often fail to report the findings in the computer-generated diagnostic statement. 1,2 For this reason, clinicians should not rely on computer algorithms; they should have an independent method of assessing the QT interval so as not to miss this critical diagnosis.

The “rule of thumb” or “Half the RR” (Half-RR) rule is one such option. It estimates the QT segment to be prolonged if it occupies greater than one-half the R wave to R wave interval, and is a favored clinician screening tool due to its ease of use. Other options for clinician-driven QT interval assessment are more laborious. QT correction formulas require the user to measure the raw QT interval, then calculate a “corrected” QT (QTc) to determine QT prolongation. QTc formulas have their own associated errors, and no QTc formula is clearly superior. 3–7 The Chan QT nomogram offers an outcome-oriented assessment of the QT interval but requires the user to plot the raw QT interval against heart rate to determine whether the patient is at risk of TdP. 8 This clinically-oriented approach is promising but has not been prospectively validated and requires additional analysis on the part of the clinician, which limits its widespread use.

In prior studies, the Half-RR rule has performed poorly when compared to various QTc formulas and the QT nomogram. 9 However, without a simple screening tool like the Half-RR rule, clinicians are likely to rely more heavily on computer measurements that are unreliable and inaccurate. Rather than discard the Half-RR rule entirely, we aimed to assess the reliability of the commonly used Half-RR rule and find a modified, easy-to-use screening tool to more safely assess the QT interval in ED patients for prolonged QT.

## METHODS

### Graph Development and Initial Comparison

We used R software (open source, version 3.4.4) to create graphs comparing the prediction of the Half-RR rule to various common QT assessment methods, including the Chan QT nomogram and the Bazett, Fridericia, Framingham, and Hodges QTc formulas. These graphs considered all possible QT-heart rate pairs, with QT intervals ranging from 300 milliseconds (ms) to 1000 ms and heart rates ranging from 40 beats per minute (bpm) to 150 bpm. The prediction of the given QT correction method (ie, prolonged vs not prolonged QT interval) for each QT-heart rate pair was calculated and is reflected on the graph. For the QT correction formulas, a QTc of 485 ms and higher was considered prolonged. We chose this value recognizing that the upper limit of normal for QTc varies by gender and formula used. While no perfect cutoff has been established, prior studies suggest that a QTc of 485 ms is beyond the upper limit of normal in both genders and in all formulas used in this study. 4,7

We then created a series of agreement graphs to better identify occasions that prediction of the Half-RR rule differed from the other methods. All possible QT-heart rate pairs were plotted and identified as “prolonged” or “not prolonged” according to the correction method used in that graph. We then compared the Half-RR graph to each of the various other QT assessment methods to highlight areas of agreement and disagreement between the Half-RR rule and that particular method.

**Population Health Research Capsule**

What do we already know about this issue?

The “Half the RR” (Half-RR) rule is a popular screening tool for prolonged QT, but it performs poorly compared to other QT assessment methods.

What was the research question?

To identify the pitfalls of the Half-RR rule and find a modified screening tool that safely assesses for prolonged QT.

What was the major finding of the study?

Adding a fixed QT cutoff of 485 milliseconds in bradycardia increases the sensitivity of the Half-RR rule, creating a safer screening tool.

How does this improve population health?

Using this modified rule will enhance screening for prolonged QT and improve the identification of patients at acute risk of torsades de pointes and sudden cardiac death.

### Development of New Screening Rules

After understanding the problem areas for the Half-RR rule, we then considered various modifications to improve the rule of thumb as a screening tool for clinicians. We created several new screening rules in an attempt to improve the sensitivity of the rule of thumb in bradycardia without compromising the specificity at higher heart rates.

### Data analysis

We analyzed the test characteristics of the new screening rules using standard diagnostic statistics and calculated using R statistical computing software, version 3.4.4.

## RESULTS

**Figure 1:**
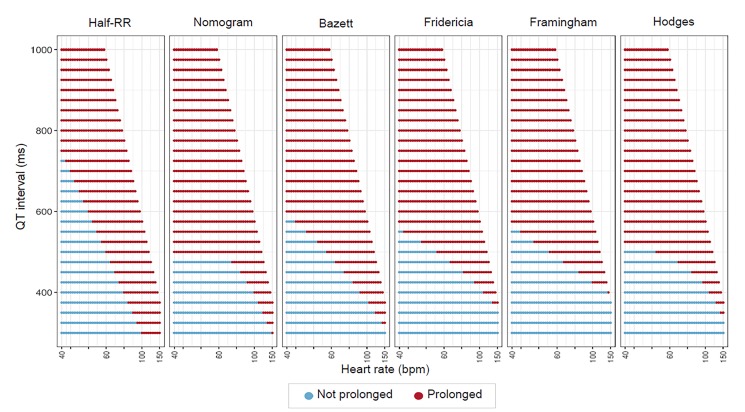
Prediction of various QT correction methods. This graph shows the predictions of each QT correction method (ie, prolonged vs not prolonged QT interval) for various QT-heart rate pairs. The Half-RR rule differs significantly from the remainder of the methods. In bradycardia, the Half-RR rule labels fewer QT intervals as “prolonged” as compared to the other methods. In tachycardia, the Half-RR rule labels more QT intervals as “prolonged.” *ms*, milliseconds; *RR*, R wave to R wave interval; *bpm*, beats per minute.

The performances of the various QT assessment methods over a range of QT interval and heart rate pairs is depicted in Figure 1.

The Half-RR rule is notably different from the other graphs, but most closely mimics the other QT correction methods between heart rates of 60–100 bpm. At heart rates below 60 bpm, the Half-RR rule labels too many QT intervals as normal, thus producing more false negatives. In tachycardia, the Half-RR tends to label too many QT intervals as prolonged, and thus has more false positives.

**Figure 2:**
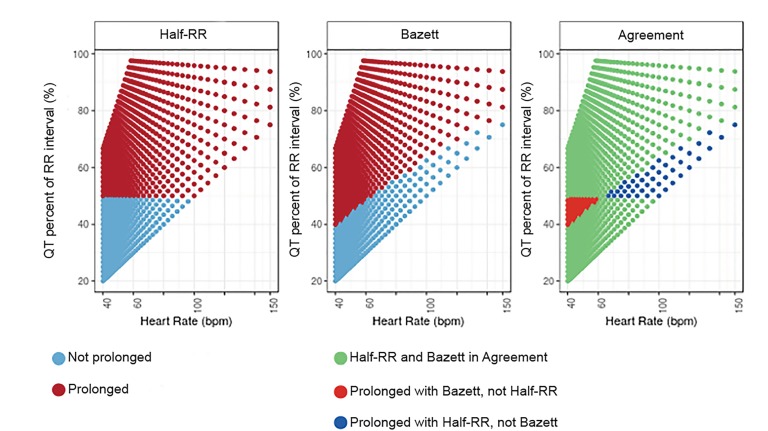
Agreement Between the Half-RR and Bazett’s Formula. The left and center graphs show the prediction of the Half-RR rule and the Bazett correction method for various QT-heart rate pairs, showing the QT interval as a percent of the RR interval on the y-axis. The right graph shows the areas of agreement and disagreement between the Half-RR rule and Bazett correction method, showing that the Half-RR rule is less conservative than Bazett in bradycardia, but more conservative at higher heart rates. *bpm*, beats per minute.

**Figure 3:**
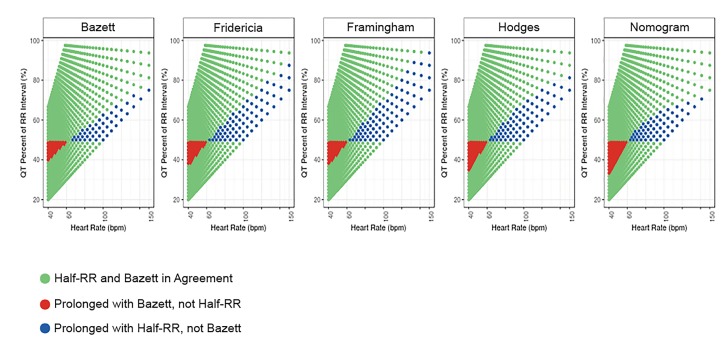
Areas of agreement and disagreement among the Half-RR rule and the remaining QT correction methods. Red areas represent occasions when the Half-RR rule is less conservative than the listed QT correction method. These instances only occur in bradycardia. *RR*, R wave to R wave interval; *bpm*, beats per minute.

Figures 2 and 3 highlight the areas of agreement and disagreement between the Half-RR rule and other QT assessment methods and also support this assessment. At heart rates between 60–66 bpm, the Half-RR rule is accurate as compared to the other methods. Below 60 bpm, the Half-RR rule often failed to note prolonged QT as indicated by all other methods. By contrast, above 66 bpm,the Half-RR rule was overly conservative. At 96 bpm, all four formulas consider a QT stretching 60% of the RR interval to be not prolonged, indicating that at high heart rates, the Half-RR rule produces many false positives.

**Figure 4:**
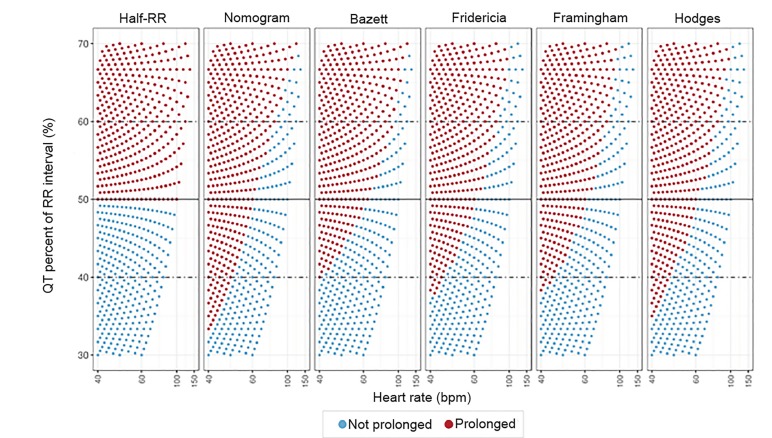
A perfect formula “rule of thumb” based on percentage is impossible. Changing the percentage in the rule of thumb to 60% (ie, raising the horizontal line of demarcation) increases the specificity at higher heart rates but increases the false negatives at low and normal heart rates. Lowering the percentage to 40% (ie, lowering the line of demarcation) would make the screening tool more sensitive in bradycardia but would result in many more false positives at normal and high heart rates. *RR*, R wave to R wave interval; *bpm*, beats per minute.

In Figure 4, we considered whether changing the percentage from 50% of the RR interval to a higher or lower percentage would result in a better rule of thumb. Lowering the percentage to 40% of the RR interval produces far too many false positives at higher heart rates. Raising the percentage to 60% of the RR interval produces far too many false negatives at lower heart rates.

Keeping in mind our goal of creating a screening rule for clinicians to use to routinely assess QTc prolongation by mental math, we developed several new rules of thumb aimed at improving the sensitivity of the rule in bradycardia without sacrificing specificity at higher heart rates. The proposed rules (Table 1) focus on percentages and fixed cutoffs so that they would be easy to calculate and remember.

**Figure 5:**
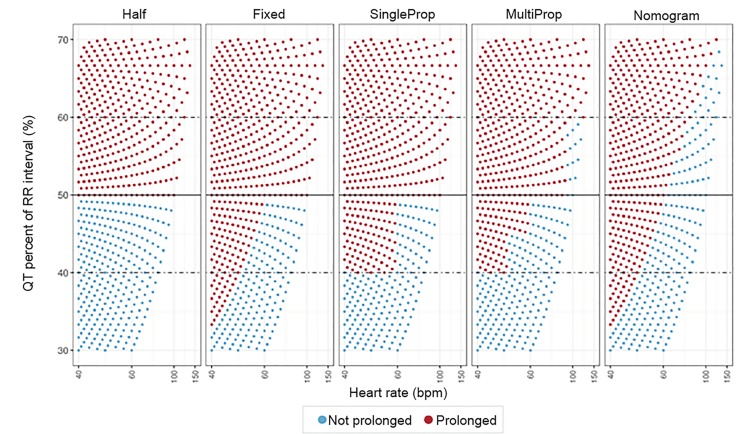
Comparison of the performance of various, newly proposed “rules of thumb.” The proposed “fixed” rule most closely mimics the QT nomogram and improves the sensitivity of the Half-RR rule in bradycardia.

The proposed screening rules were compared to the QT nomogram given its promising data and clinically-oriented focus. Figure 5 demonstrates how the increasingly complex rules successively fill in the additional area where the traditional half-RR rule of thumb disagrees with the nomogram.

The “fixed” rule, a combination of the Half-RR rule with a fixed cut-off of 485 ms in bradycardia, most closely mimics the QT nomogram. The sensitivity of the unmodified Half-RR rule for detecting QTc prolongation, using the nomogram as a reference standard, is 84.2% (95% confidence interval [CI], 81.5–86.9%). The addition of the fixed cutoff of 485 ms in bradycardia raises the sensitivity to 100% (99.5–100.0%). The single and multiple proportional rules have 96.1% (94.7–97.5%) and 95.3% (93.7–96.8%) sensitivity. The specificity of these rules ranges from 75.4% to 80.3%. Table 2 presents the full test characteristics of each rule.

## DISCUSSION

Our analysis shows consistently poor test characteristics of the Half-RR rule as compared to other methods of QT interval assessment. In bradycardia, the Half-RR rule consistently misses cases of prolonged QT as identified by all other QT correction methods. At normal and elevated heart rates, the Half-RR rule produces many more false positives as compared to other QT correction methods. This is consistent with prior research, which has shown the Half-RR rule to have a poor sensitivity at heart rates below 60 bpm, but 100% sensitivity and approximately 50% specificity with heart rates above 60 bpm. 9

The Half-RR rule is used primarily as a screening tool; thus, a low sensitivity in any clinical context is problematic. The poor sensitivity in bradycardia is of particularly serious concern given that patients are most clinically at-risk of TdP when they are bradycardic due to the pause-dependent TdP phenomenon. Lowering the percentage used in the rule of thumb was not an acceptable solution to this problem, as doing so negatively impacted the specificity of the rule. Of the newly considered modified rules of thumb, the “fixed” rule adds a simple modification to the Half-RR rule to resolve the poor sensitivity in bradycardia. For heart rates below 60 bpm, the raw QT is declared prolonged when above 485 ms, achieving excellent sensitivity (100%, CI, 99.5–100.0%) without unduly decreasing specificity.

At normal and elevated heart rates, our analysis shows that the Half-RR rule is more conservative than other QT assessment methods and produces many more apparent false positives. The new “fixed” RR rule does not address this issue. Thus, if the “fixed” RR rule deems a QT interval “prolonged” at any heart rate above 66 bpm, the clinician should proceed with formal measurement and risk assessment based on the QT nomogram or one of the correction formulas.

The proposed “fixed” RR rule is simple to use and remember. It is a safe and realistic initial screening tool for QT prolongation for emergency clinicians. Using this screening tool should improve recognition of prolonged QT in bradycardia in the ED and assist clinicians in safely “ruling-out” prolonged QT at normal and elevated heart rates.

## LIMITATIONS

There are several limitations to this discussion. First, the Half-RR rule ideally would be evaluated by comparing it to a gold-standard formula or nomogram that has been carefully calibrated against a large database with mortality as the outcome. Such a gold standard does not exist. The existing QT correction rules were not derived with mortality outcomes in separate validation samples, although the Bazett correction has been used to correlate long QTc with long-term, but not short-term, outcomes. 9 Instead, we compared the Half-RR method to each of the four formulas and the QT nomogram, effectively substituting usual care for the unattainable gold standard.

Second, these measures depend on the population values. While sensitivity and specificity do not vary with population prevalence in theory, in practice they seem to do so. 10 Since we have arbitrarily generated a population of values, these values may change slightly if we knew that particular ratios of RR intervals to QT intervals were more common. Still, in the absence of data on prevalence of RR and QT pairs in the ED, it is difficult to improve upon this strategy of comparing to the existing – and more complicated – rules.

Finally, the above discussion implies that the variation of QT interval across heart rates is alike in all individuals. However, a substantial body of research shows that there is great interindividual variability and even intrasubject variability. 3,11,12 The most accurate way to know a patient’s true corrected QT at a given heart rate is to measure and plot the individual patient’s QT interval over a range of heart rates. Of course, this task is not realistic in the ED setting. The discussion and strategies offered above provide a reasonable and more realistic approach to QT interval assessment without highly personalized patient data.

## CONCLUSION

Recognizing and addressing prolonged QT intervals is critical in the ED. Accurately identifying patients with dangerously prolonged QT intervals allows emergency clinicians to intervene on patients who are at acute risk of TdP and to avoid discharging patients at risk of sudden death. There are many complexities in measuring and correcting the QT interval, and, unfortunately, computer algorithms cannot be relied upon for accurate QT measurement and correction. When the heart rate is above 60 bpm, the Half-RR rule is a conservative screening tool and may be safely used. In bradycardia, the Half-RR rule is prone to false negatives and should not be used. Instead, a fixed cutoff of 485 ms is likely a better measure, but further validation is required.

## Figures and Tables

**Table 1:** Proposed new “rules of thumb.”

Fixed	Half-RR rule above 60 beats per minute (bpm), fixed cutoff of 485 below 60 bpm
Single Proportional	Half-RR rule above 60 bpm, 40% RR below 60 bpm
Multiple Proportional	60% RR above 90 bpm, Half-RR rule above 60 bpm, 45% RR below 60 bpm, 40% RR below 50 bpm

**Table 2:** Diagnostic test characteristics of the proposed rules of thumb compared to the Chan nomogram.

	Sensitivity (%)	Specificity (%)	Positive predictive value (%)	Negative predictive value (%)	Positive likelihood ratio	Negative likelihood ratio
Half-RR	84.2	80.3	91.1	67.8	4.3	0.2
Fixed	100	80.3	92.4	100	5.1	0
SingleProp	96.1	75.4	90.1	90.0	3.9	0.1
MultiProp	95.3	84.1	93.5	88.0	6.0	0.1
